# Personalized Care in Opioid Use Disorder maintained on Buprenorphine

**DOI:** 10.1192/j.eurpsy.2022.2116

**Published:** 2022-09-01

**Authors:** H. Elarabi, J. Marsden, H. Alghafri, A. Adem, A. Lee

**Affiliations:** 1 National Rehabilitation Center, Technical Office / Surveillance And Policy, Abu Dhabi, United Arab Emirates; 2 King’s College London, Institute Of Psychiatry, Psychology And Neuroscience, London, United Kingdom; 3 National Rehabilitation Center, Nrc, Abu Dhabi, United Arab Emirates; 4 Khalifa University, Faculty Of Medicine, Abu Dhabi, United Arab Emirates; 5 University of Aberdeen, Institute Of Applied Health Sciences, Aberdeen, United Kingdom

## Abstract

**Introduction:**

Effectiveness of buprenorphine (BUP) is moderated by medication misuse diversion and non-adherence, and poor retention and high cost. Contingency Managment has added benefit to BUP with Take-home doses as the most preferred reward by Opioid Use Disorder patients.

**Objectives:**

Examine the clinical effectiveness of a novel incentivised medication adherence and abstinence monitoring to enable contingent access to increasing BUP take-home doses.Explore associations with opioid use and retention. Contrast characteristics of polysubstance abusers (PSA) and response to BUP, with single opioid users.

**Methods:**

Two-arm, pragmatic, 16-week outpatient RCT of BUP maintenance.Takehome doses were provided as stepped-approach upto 4-weeks contingent of abstinence (UDS) and adherence according to Therapeutic Drug Monitoring-TDM. Primary outcome and secondard outcomes were % negative UDS for opioids anx retention, respectively. -

**Results:**

Opioid % negative UDS was 76.7% (SD 25.0%) in I-AAM versus 63.5% (SD 34.7%) in TAU (13.3%; 95% [CI] 3.2%–23.3%; Cohen’s d 0.44; 95% CI 0.10–0.87). In I-AAM, 40 participants (57.1%) were retained versus 33 (46.4%) in TAU [OR: 1.54; 95% CI 0.79–2.98). PSA (73.7%, n=104) and carisprodol use increases non-fatal overdose (OR) 3.83, 95% CI 1.25 to 11.71) and 5.31, 95% CI 1.92 to 14.65], respectively. Opioid and non-opioid UDS are positively associated. BUP elimination rate (BUP-EL.R) predicts 26.5% to 65% of negative opioid UDS [Beta - 89.95, 95% CIl -154.20 to -25.70, R2 0.22]. Family enagement increases retention by 3-fold.

**Conclusions:**

BUP + incentivised TDM for contingent access to increasing take-home doses increased abstinence. BUP-EL.R seems promising in BUP treatment precision and BUP is clinically valuable in polysubstance abusers.Engaging family enhances retention.

**Disclosure:**

No significant relationships.

